# Complete Genome Sequence of an Enterovirus Type C116 Strain Recovered from Urban Sewage and Determined by Deep Sequencing

**DOI:** 10.1128/mra.00226-23

**Published:** 2023-04-11

**Authors:** Maxime Bisseux, Audrey Mirand, Jonathan Colombet, Jean-Luc Bailly, Cécile Henquell

**Affiliations:** a CHU Clermont-Ferrand, 3 IHP, Centre National de Référence des Entérovirus et Parechovirus, Laboratoire de Virologie, Clermont-Ferrand, France; b Université Clermont Auvergne, LMGE CNRS 6023, UFR de Médecine et des Professions Paramédicales, Clermont-Ferrand, France; Queens College Department of Biology

## Abstract

Wastewater surveillance allowed the detection of an enterovirus (EV) type rarely reported in clinical settings. We detected an EV-C116 strain in a wastewater sample in France and characterized its complete genome. This virus was genetically closely related to African strains but distantly related to the only complete genome previously described.

## ANNOUNCEMENT

*Enterovirus* type C116 (EV-C116) belongs to the family *Picornaviridae* and the species *Enterovirus* C. The only whole-genome sequence previously available (GenBank accession number JX514942.1) was obtained from a stool specimen from a Russian child with diarrhea in 2010 (1). Since then, EV-C116 has mainly been detected through urban sewage monitoring.

From October 2014 to October 2015, we conducted prospective wastewater-based surveillance of enteroviruses at a sewage treatment plant serving a catchment population of 300,000 (Clermont-Ferrand, France) (2, 3). Total nucleic acids were extracted from 200 μL of concentrated wastewater using the NucliSENS EasyMag platform to an elution volume of 50 μL (bioMérieux, Marcy l’Etoile, France). Reverse transcription was performed as indicated by the manufacturer with superscript IV reverse transcriptase (Invitrogen, Cergy-Pontoise, France) and the reverse primer polyT3NC_EVC_R1 (Table 1). Amplification of the complete genome was performed using Platinum SuperFi-I (Invitrogen) and the primers IFT7_EVC_S1, IFT7_EVC_S2 and 3NC_EVC_R1, as indicated by the manufacturer, with a hybridization temperature of 67.5°C and an elongation time of 270 s. The PCR product was purified using 0.45× AMPure PacBio beads. A SMRTbell library was prepared using the DNA template prep kit v1.0, following the procedure for preparing amplicon libraries using PacBio barcoded adapters for multiplex single-molecule real-time (SMRT) sequencing. The library quality was assessed using a fragment analyzer system (Agilent Technologies) and quantified (Qubit fluorimeter and Qubit double-stranded DNA [dsDNA] high-sensitivity [HS] reagent assay kit; Life Technologies). The library was sequenced over 20 h with the Sequel I system using the binding kit v3.0, v4 primer, and PacBio SMRT cells v2.0 (Pacific Biosciences, Menlo Park, USA). The run provided 665,105 raw reads, with an average length of 41,090 nucleotides (nt) and an average insert *N*_50_ size of 7,870 nt.

**TABLE 1:** Primers used for the reverse transcription, amplification, and sequencing of the complete EVC116 genome

Primer designation	Primer sequence (5′–3′)	Orientation
PolyT_3NC_EVC_R1	TTTTTTTTTTTTTTTTTTTTTTTTTTCTCCGAATYAAAGAAAAATTTACCC	Reverse
IFT7_EVC_S1	GACAGCTTATCATCGTAATACGACTCACTATAGGGTTAAAACAGCYTGDGGGTTGTTC	Forward
IFT7_EVC_S2	GACAGCTTATCATCGTAATACGACTCACTATAGGGTTAAAACAGCTCTGGGGTTGTTC	Forward
3NC_EVC_R1	TCTCCGAATYAAAGAAAAATTTACCCCTAC	Reverse

The raw PacBio reads were assembled using SMRT Link software v6.0.1 (Pacific Biosciences) (4). Consensus circular sequences were obtained using the pbsmrtpipe tool and then filtered and demultiplexed using the Lima tool (5). Default parameters were used for all software unless otherwise specified. A final consensus sequence, generated based on an 80% similarity threshold, was compared to the EV-C116 genome found under GenBank accession number JX514942.1 to determine its completeness. The new full-length EV-C116 genome (7,385 nt) was designated EV-C116_Wastewater_18/08/15_CFD_France and determined, based only on PacBio sequencing, to have a GC content of 43.3%.

**FIG 1:**
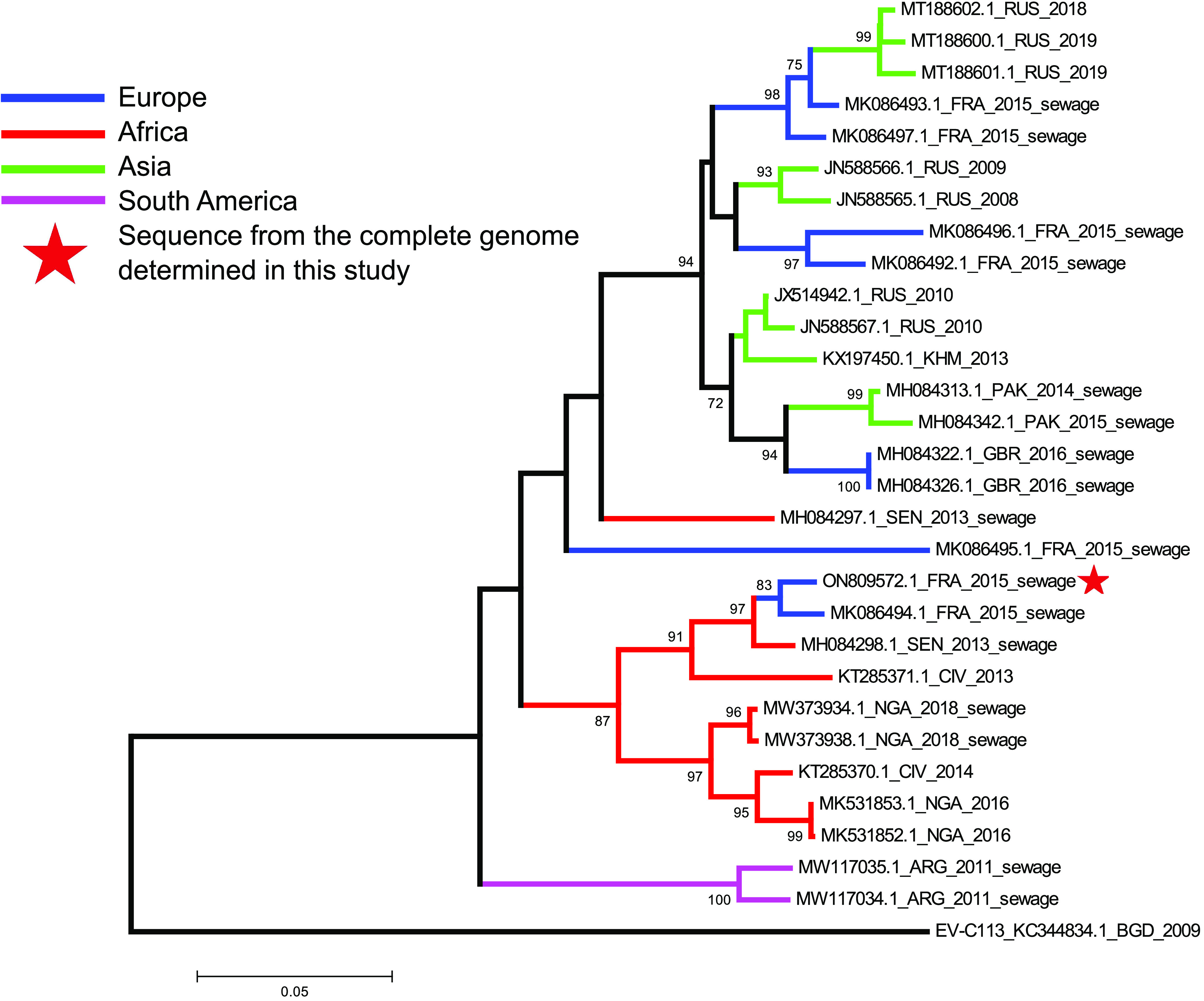
Genetic analysis of the EV-C116 genome. The phylogenetic tree was based on the alignment of partial EV-C116 VP1 gene sequences (340 nt) available at GenBank using MEGA5 software, with the Tamura Nei method, using 1,000 bootstrap replicates. The country of each strain is indicated with the ISO 3166-1 alpha-3 code. Strains isolated from wastewater are labeled as such.

Phylogenetic reconstruction based on partial VP1 sequences was performed between the new sequence and the 16 publicly available homologous sequences (Fig. 1). The genetic relationships between sequences from strains detected in wastewater samples from across Europe, Africa, South America, and Asia attest to the worldwide detection of EV-C116. This pattern contrasts with the fact that very few symptomatic patients have been reported (1, 6). The phylogenetic tree inferred from the partial VP1 gene sequences showed distinct clusters. Four of the seven sequences sampled in France in 2015 clustered with sequences detected between 2008 and 2019 in Russia. Two other sequences (including that of the genome in this study) were closely related to sequences from strains collected between 2013 and 2018 in sewage from Senegal and Nigeria and in stool specimens from healthy individuals living in Ivory Coast (7–9).

### Data availability.

This whole-genome sequence has been deposited at GenBank under the accession number ON809572. The version described in this paper is the first version. The raw data are available in the Sequence Read Archive (SRA) under accession number SRR23605948, BioProject accession number PRJNA938282, and BioSample accession number SAMN33430181.
